# Autoantibodies Targeting Vinculin Reveal Novel Insight into the Mechanisms of Autoimmune Podocytopathies

**DOI:** 10.34133/research.0722

**Published:** 2025-06-03

**Authors:** Hanyan Meng, Dongjie Wang, Chen Zheng, Chao Zhou, Xinrui Mao, Jinglan Gu, Xiaohui Qiao, Fei Liu, Jingjing Wang, Haidong Fu, Jianhua Mao, Qing Ye

**Affiliations:** ^1^ Department of Nephrology, Children’s Hospital, Zhejiang University School of Medicine, National Clinical Research Center for Child Health, National Children’s Regional Medical Center, Hangzhou 310052, China.; ^2^ Department of Nephrology, Women and Children’s Hospital of Ningbo University, Ningbo 315000, China.; ^3^ Department of Laboratory Medicine, Children’s Hospital, Zhejiang University School of Medicine, National Clinical Research Center for Child Health, National Children’s Regional Medical Center, Hangzhou 310052, China.

## Abstract

**Background:** Emerging evidence suggests that autoantibodies targeting podocytes are potential contributors to idiopathic nephrotic syndrome (INS); however, the specific mechanisms remain unclear. This study aims to explore the pathogenic role and underlying mechanisms of anti-vinculin autoantibodies in INS. **Methods:** Serum anti-vinculin autoantibody levels detected by protein microarray and clinical data were compared among INS patients (*n* = 147), healthy individuals (*n* = 84), and patients with other kidney or immune diseases (*n* = 100 of each disease). Immune-mediated mouse models were established to verify the pathogenicity of anti-vinculin autoantibodies. Mouse urine was monitored for urine protein levels, while immunofluorescence, pathological staining, and electron microscopy assessed kidney pathological and ultrastructural changes. Transcriptome sequencing of mouse kidney tissues was performed to investigate the key molecular mechanisms and signaling pathways involved in kidney injury post-immunization. **Results:** Anti-vinculin autoantibody levels were specifically elevated in INS patients, with a 54.42% positivity rate, correlating with urinary albumin, serum albumin, cholesterol, and CD19 levels. The average anti-vinculin autoantibody levels dropped markedly in pediatric INS patients during remission. Mouse experiments revealed that injecting anti-vinculin antibodies or recombinant vinculin protein induced proteinuria and podocyte injury in the immunized mice, and the renal phenotype closely resembled the pathological characteristics of minimal change disease. Transcriptome sequencing of renal tissues revealed up-regulation of inflammation, immune responses, cytokine activities, and B cell activation pathways in the immunized mice, while cytoskeleton-related functions were down-regulated. **Conclusions:** Autoantibodies targeting vinculin act as pathogenic autoantibodies in INS and hold potential value for diagnosing and monitoring INS progression.

## Introduction

Idiopathic nephrotic syndrome (INS), characterized by massive proteinuria and hypoproteinemia, is one of the most common glomerular diseases in children and is considered an autoimmune-mediated podocytopathy [1,2]. Diagnosis usually requires a combination of clinical symptoms, laboratory tests, and kidney biopsies assessed with immunofluorescence, light microscopy, and electron microscopy [3]. Minimal change disease (MCD), the predominant pathological condition in pediatric INS, is characterized by minimal alterations observable under light microscopy. However, it has significant diffuse foot process effacement and slit diaphragm disruption observed via electron microscopy [2,4]. Traditionally, renal biopsies from MCD patients lack immunoglobulin deposits or electron-dense material, leading to an incomplete understanding of their immunopathogenesis [5]. This uncertainty often results in nonspecific therapies, risking complications such as drug resistance or adverse effects. In recent decades, substantial progress has been made in exploring the mechanisms underlying autoimmune podocytopathies, yet critical questions remain unanswered.

Recent findings have shed new light on the potential role of autoantibodies in the pathogenesis of INS. For instance, B cell-targeted therapies have demonstrated efficacy in INS patients with frequent relapses or steroid dependence, suggesting that humoral immunity plays a critical role in disease activity. Several studies have identified specific autoantibodies associated with INS [2,6–11]. Watts et al. [7] discovered anti-nephrin autoantibodies in a subset of adults and children with MCD during disease activity, providing initial evidence for a potential autoantibody-mediated mechanism. Shirai et al. [12] further corroborated these findings by showing that serum levels of anti-nephrin autoantibodies were considerably elevated in patients with recurrent focal segmental glomerulosclerosis (FSGS) post-kidney transplantation and subsequently decreased as the disease went into remission. Building on these observations, Hengel et al. [2] conducted a multicenter study to investigate the link between anti-nephrin autoantibodies and disease activity and developed a mouse model using recombinant murine nephrin for active immunization. These studies collectively underscore the importance of anti-nephrin autoantibodies in the pathophysiology of INS.

In addition to autoantibodies targeting nephrin, other potential targets have been proposed. Raglianti et al. [6], through high-resolution microscopy of kidney biopsies and enzyme-linked immunosorbent assay (ELISA), suggested that autoantibodies present in the sera of patients with INS are directed not only against nephrin but also against other podocyte-associated antigens. Jamin et al. [8] reported that autoantibodies against ubiquitin carboxy-terminal hydrolase L1 (UCHL1) are closely related to the recurrence of INS, further expanding the repertoire of potential autoantibody targets. Similarly, Hada et al. [9] demonstrated that immunizing mice with Crb2, a protein expressed in podocytes, induced anti-Crb2 autoantibodies, resulting in proteinuria and foot process effacement. Raglianti et al. [13] identified anti-podocin and anti-kirrel1 autoantibodies as highly specific biomarkers for active INS. These findings highlight the complexity of the autoimmune response in INS, revealing that multiple autoantibodies can coexist and target various antigens in the same patient, broadening the spectrum of autoimmune podocytopathies.

Our team has been focusing on the immunopathogenesis and immunotherapy of INS in children for several years. We previously elucidated a critical role for B cells in the pathogenesis of INS [14,15]. On the basis that B cells primarily exert their effects through the secretion of autoantibodies [16,17], we identified a range of autoantibodies in the serum of INS patients [16–18] and proposed the concept of “autoimmune podocytopathies” [19]. This study aims to determine the prevalence and clinical implications of anti-vinculin autoantibodies in INS as well as investigate their role and pathogenic mechanisms . Vinculin is a key protein critical for podocyte cytoskeletal integrity, which plays an essential role in maintaining cellular structure and function [20,21]. Specifically, the podocyte-specific knockout of *Vcl* in mice leads to albuminuria and podocyte injury [22]. Therefore, we hypothesize that anti-vinculin autoantibodies alter the structure and function of vinculin, resulting in podocyte injury and disruption of the filtration barrier. A thorough investigation of anti-vinculin autoantibodies could advance our understanding of their roles and mechanisms in terms of clinical implications, pathogenic mechanisms, and potential as therapeutic targets in INS.

## Results

### Elevated anti-vinculin autoantibodies in INS patients correlate with disease activity

**Fig. 1.**
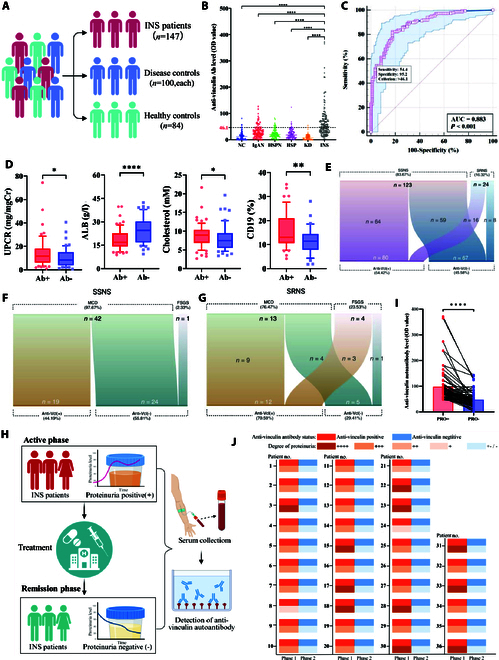
Anti-vinculin autoantibodies in the serum of pediatric patients with INS. (A) Study cohort and included patients. (B) Comparison of anti-vinculin autoantibody levels in the serum of INS patients and controls. NC, normal control group; IgAN, IgA nephropathy; HSPN, Henoch–Schönlein purpura nephritis; HSP, Henoch–Schönlein purpura; KD, Kawasaki disease. (C) ROC curve corresponding to the levels of anti-vinculin in INS patients and healthy controls. (D) Comparison of the clinical data of the anti-vinculin autoantibody-positive and anti-vinculin autoantibody-negative groups. UPCR, urinary protein/creatinine ratio; ALB, serum albumin. (E) Alluvial charts of patient sensitivity to glucocorticoid therapy (above) and anti-vinculin antibody status (below). (F and G) Alluvial charts of pathology patterns at kidney biopsy (above) and anti-vinculin antibodies status (below) in patients with SSNS and SRNS. (H) Longitudinal self-pairing analysis strategy. (I) Longitudinal paired analysis of anti-vinculin autoantibody levels and proteinuria levels. (J) Visualization of the correlation between anti-vinculin autoantibodies and the longitudinal disease course during the acute phase and remission.

A retrospective study was conducted among INS patients (*n* = 147), healthy individuals (*n* = 84), and patients with other kidney or immune diseases (*n* = 100 for each disease). The level of anti-vinculin autoantibodies was significantly elevated in INS patients during the active phase compared with healthy controls and disease controls, with a positivity rate of 54.42% (Fig. 1A and B). The threshold for anti-vinculin autoantibody positivity was set at 46.1, based on the 95th percentile titer observed in the healthy control population. Receiver operating characteristic (ROC) curve analysis revealed a high area under the curve (AUC) of 0.882 (*P* < 0.001), with a sensitivity of 54.4% and specificity of 95.2%, when the criterion was set above 46.1 (Fig. 1C). We divided the cohort of 147 INS patients into 2 groups on the basis of the positive cutoff (46.1). Patients who were positive for anti-vinculin autoantibodies (*n* = 80) predominantly exhibited severe nephrotic syndrome (Fig. 1D and Table), characterized by significantly higher urinary protein/creatinine ratio, lower serum albumin levels, and increased serum cholesterol and CD19 levels. Most pediatric INS patients respond well to glucocorticoid treatment, regardless of their autoantibody status. Specifically, 64 of 80 (80%) patients in the anti-vinculin autoantibody-positive group and 59 of 67 (88.1%) in the negative group were classified as having steroid-sensitive nephrotic syndrome (SSNS), as shown in Fig. 1E. Renal biopsy reports were reassessed for patients with available renal biopsy reports (*n* = 69). Among these, 60 (86.96%) presented the most common podocytopathy pattern in children: MCD and FSGS. Among the 69 patients, 43 were diagnosed with SSNS, and 17 with steroid-resistant nephrotic syndrome (SRNS). Positive anti-vinculin autoantibodies were detected in 19 of the 43 SSNS patients (44.19%) and in 12 of the 17 SRNS patients (70.59%), as shown in Fig. 1F and G. Among the 80 INS patients positive for anti-vinculin autoantibodies, serum samples were available for both the active and remission phases in 69 patients, enabling a self-paired comparison of antibody levels before and after proteinuria remission, as shown in Fig. 1H. The analysis revealed a statistically significant reduction in anti-vinculin autoantibody levels in the remission period (Fig. 1I). Furthermore, among the tested individuals, 36 of the 69 patients (52.17%) transitioned to completely antibody-negative status in remission, which coincided with the cessation of proteinuria after treatment (Fig. 1J).

**Table.** Clinical characteristics of INS patients

	Total (*n* = 147)	Anti-vinculin positive (*n* = 80)	Anti-vinculin negative (*n* = 67)
Female, *n* (%)	46 (31.29%)	30 (37.50%)	16 (23.88%)
Male, *n* (%)	101 (68.71%)	50 (62.50%)	51 (76.12%)
Age (year)	5.0 (3.00, 11.00)	4.0 (3.00, 9.00)	7.0 (4.00, 11.00)
UPCR (mg/mgCr)	10.91 (4.75, 16.70)	11.63 (6.07, 18.07)	8.315 (3.85, 15.10)
ALB (g/l)	18.4 (15.53, 27.10)	16.80 (14.30, 22.60)	24.50 (16.85, 30.25)
Triglyceride (mM)	2.57 (1.83, 3.95)	2.67 (2.00, 4.10)	2.335 (1.56, 3.61)
Cholesterol (mM)	8.56 (6.15, 10.11)	8.92 (6.86, 10.37)	7.465 (5.50, 9.48)
eGFR (ml/min/1.73 m^2^)	134.20 ± 32.49	134.50 ± 35.83	133.90 ± 28.17
Renal biopsy, *n* (%)	68 (46.26%)	36 (45.00%)	32 (47.76%)
MCD, *n* (%)	55 (80.88%)	28 (77.78%)	27 (84.38%)
FSGS, *n* (%)	5 (7.35%)	3 (8.33%)	2 (6.25%)
MsPGN, *n* (%)	6 (8.82%)	4 (11.11%)	2 (6.25%)
MN, *n* (%)	2 (2.94%)	1 (2.78%)	1 (3.12%)
Serum anti-vinculin autoantibody level	49.70 (29.00, 79.00)	74.70 (58.45, 106.50)	28.20 (17.30, 36.00)

UPCR, urine protein/urine creatinine ratio; ALB, serum albumin; eGFR, estimated glomerular filtration rate; MCD, minimal change disease; FSGS, focal segmental glomerulosclerosis; MsPGN, mesangial proliferative glomerulonephritis; MN, membranous nephropathy

**Fig. 2.**
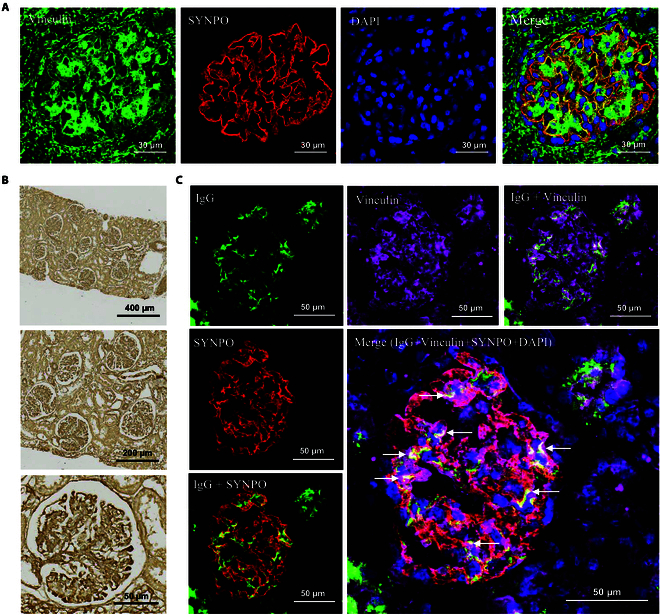
Expression and localization of vinculin and anti-vinculin autoantibodies in human kidneys. (A) Immunofluorescence staining of vinculin in human glomerular podocytes. Magnification: 80× (scale bar, 30 μm). (B) Immunohistochemical staining of vinculin in human kidney tissue. Magnifications: 10× (scale bar, 400 μm), 20× (scale bar, 200 μm), and 60× (scale bar, 50 μm). (C) Immunofluorescence visualization of IgG deposition in kidney biopsies from INS children with high serum levels of anti-vinculin autoantibodies. Magnification: 63× (scale bar, 50 μm).

To explore the role of vinculin autoantibodies in INS, we analyzed vinculin expression and localization in human kidneys using normal renal tissue adjacent to renal tumors. Immunofluorescence confirmed its presence in glomerular podocytes through colocalization with Synaptopodin (SYNPO), a podocyte-specific marker (Fig. 2A). Immunohistochemistry revealed widespread vinculin expression, with higher levels in glomeruli than in tubules (Fig. 2B). To further detect anti-vinculin autoantibodies in kidney biopsies from INS patients, we performed immunofluorescence staining on renal tissue sections from patients positive for serum anti-vinculin autoantibodies. The staining revealed focal deposition of immunoglobulin G (IgG) within the renal parenchyma, demonstrating colocalization with podocytes and vinculin protein (Fig. 2C), whereas no IgG deposition was detected in the control groups (Fig. S2).

### Induction of proteinuria and podocyte injury in mice via anti-vinculin antibody injection

**Fig. 3.**
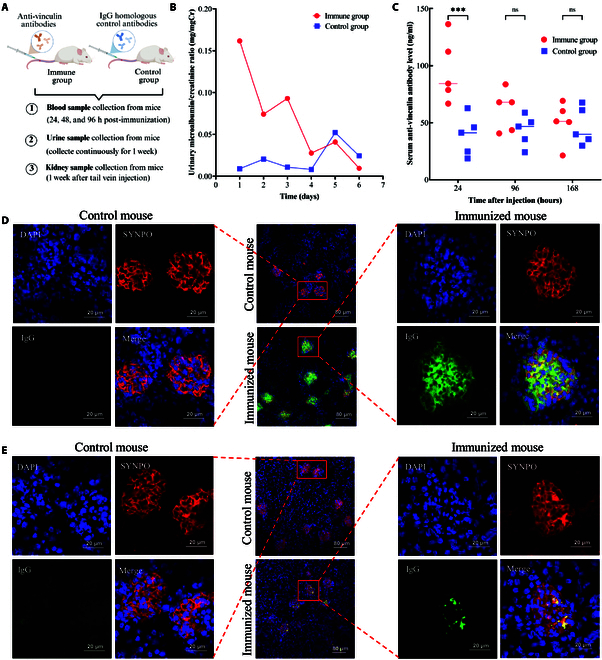
Effects of anti-vinculin antibody injection on renal function and IgG deposition in mice. (A) Tail vein injection strategies for anti-vinculin antibodies in mice. (B) Urine protein/creatinine ratios of immunized mice and controls. (C) Anti-vinculin antibody levels over time post-injection. (D and E) IgG deposition in the mouse glomeruli at 24 h and 1 week after passive immunization. Magnifications: 20× (scale bar, 80 μm) and 80× (scale bar, 20 μm).

**Fig. 4.**
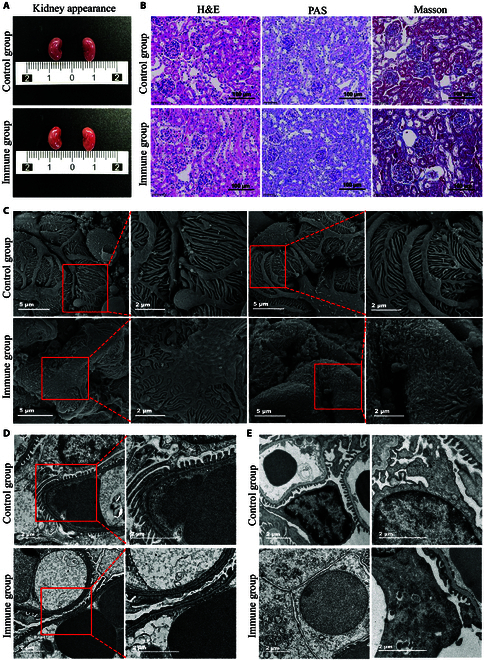
Renal morphology and histopathological analysis of passively immunized mice and control mice. (A) External appearance of the kidneys of immunized mice and control mice. (B) Pathological staining: light micrographs of H&E, PAS, and Masson staining. Magnification: 40× (scale bar, 100 μm). (C) SEM image of glomerulus-immunized mice and control mice. Magnifications: 5,000× (scale bar, 5 μm) and 12,000× (scale bar, 2 μm). (D and E) TEM images of immunized mice and control mice. Magnification: 11,000× (scale bar, 2 μm) and 22,000× (scale bar, 2 μm).

To investigate the pathogenicity of anti-vinculin autoantibodies in the kidneys, we administered anti-vinculin antibodies to mice via tail vein injection to mimic passive immunity and monitored the development of kidney damage (Fig. 3A). We continuously collected urine samples from the mice over a 1-week period and measured the level of proteinuria. During this observation period, we noted transient proteinuria in the immunized mice (Fig. 3B). We measured anti-vinculin antibody levels in mouse serum at 24, 96, and 168 h post-injection via ELISA. At 24 h, the immunized mice presented higher antibody levels than the control mice. However, these levels decreased by 96 and 168 h, leading to no significant differences between the groups at these later time points (Fig. 3C). Concurrently, at 24 h post-injection, immunofluorescence analysis revealed IgG deposition in the glomeruli of the immunized mice, whereas no IgG deposition was observed in the control mice (Fig. 3D). One week after injection, although some IgG deposition persisted in the glomeruli of the immunized mice, it was notably reduced compared with that at the 24-h mark, whereas the control group continued to show no signs of IgG deposition (Fig. 3E). No appreciable differences in kidney size or external morphology were observed between the 2 groups of mice (Fig. 4A). Histological examination via light microscopy, including hematoxylin and eosin (H&E), periodic acid–Schiff (PAS), and Masson’s trichrome staining, revealed no specific pathological alterations or discernible differences between the 2 groups (Fig. 4B). To evaluate the ultrastructures of podocytes in mice, we conducted more detailed observations of the mouse kidneys by scanning electron microscopy (SEM) and transmission electron microscopy (TEM). SEM revealed that some foot processes in the immune group were disordered, with localized fusion and damage to the foot processes. In contrast, the foot processes in the control group were neatly arranged, with a regular morphology and no evidence of fusion, as shown in Fig. 4C. TEM further revealed extensive foot process fusion in the immunized mice (Fig. 4D). Additionally, an increased number of mitochondria were observed in some podocytes, exhibiting abnormalities such as swelling, vacuolization, and crest breakage (Fig. 4E).

### Induction of proteinuria and podocyte injury in mice by vinculin immunization

**Fig. 5.**
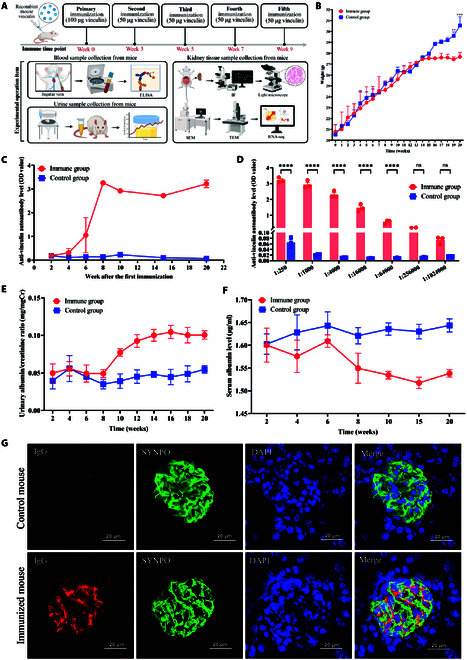
Effects of active immunization with recombinant vinculin on the immune response and renal IgG deposition in mice. (A) Strategies for active immunization with recombinant vinculin protein. (B) Weekly body weight changes of two groups of mice shown in line graphs. (C) Serum anti-vinculin autoantibody levels in mice detected by ELISA every 2 weeks post-immunization (serum diluted 1:250). (D) Serum anti-vinculin antibody titers were analyzed at 20 weeks post-immunization across different serum dilution ratios. (E) Statistical comparison of the urinary protein/creatinine ratios between immunized mice and control mice. (F) Statistical comparison of serum albumin levels between immunized mice and control mice. (G) Immunofluorescence in mice kidney shows IgG deposits (red) with podocytes (green, marked by SYNPO) in immunized mice at 20 weeks post-active immunization. Magnification: 80× (scale bar, 20 μm).

**Fig. 6.**
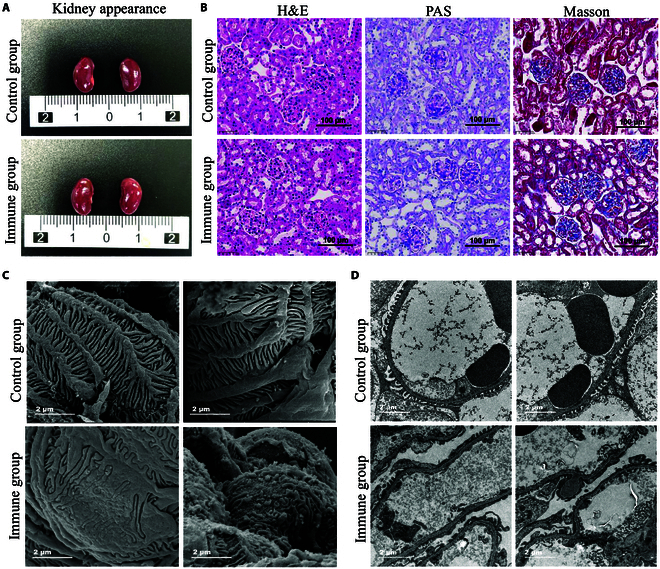
Renal morphology and histopathological analysis of actively immunized mice and control mice. (A) External appearance of the kidneys of immunized mice and control mice. (B) Pathological staining: light micrographs of H&E, PAS, and Masson staining. Magnification: 40× (scale bar, 100 μm). (C) SEM image of glomerulus-immunized mice and control mice. Magnification: 12,000× (scale bar, 2 μm). (D) TEM images of immunized mice and control mice. Magnification: 11,000× (scale bar, 2 μm).

The antibody titer decreased over time following direct immunization with anti-vinculin antibodies. To address this issue, we immunized mice by administering recombinant murine vinculin protein with Freund’s adjuvant to induce the production of anti-vinculin autoantibodies, resulting in sustained immune responses that persist for extended periods, as shown in Fig. 5A. With increasing immunization, the body weight of the immunized mice progressively fell behind the control group, with the gap widening (Fig. 5B). A marked and enduring increase in the production of anti-vinculin autoantibodies was observed in the serum of mice after active immunization, and the autoantibody level remained stable until 20 weeks (Fig. 5C and D and Fig. S3A). The presence of serum anti-vinculin autoantibodies in the immune group was also confirmed by Western blotting (Fig. S3B). As the frequency of immunization increased and the antibody titer increased, the immunized mice presented greater proteinuria and a decrease in the serum albumin concentration than the control mice (Fig. 5E and F). Immunofluorescence analysis revealed the colocalization of IgG deposits with glomerular podocytes in the renal tissue of immunized mice (Fig. 5G). Kidneys from the 2 groups of mice were comparable in size and external appearance (Fig. 6A), and histological analysis via H&E, PAS, and Masson staining revealed no overt renal pathology (Fig. 6B). Under TEM and SEM, the experimental group of mice exhibited a disarrayed configuration of foot processes, with foot process fusion and rupture, indicating podocyte injury (Fig. 6C and D).

### Transcriptome analysis of renal tissues revealed molecular features altered by vinculin immunization

**Fig. 7.**
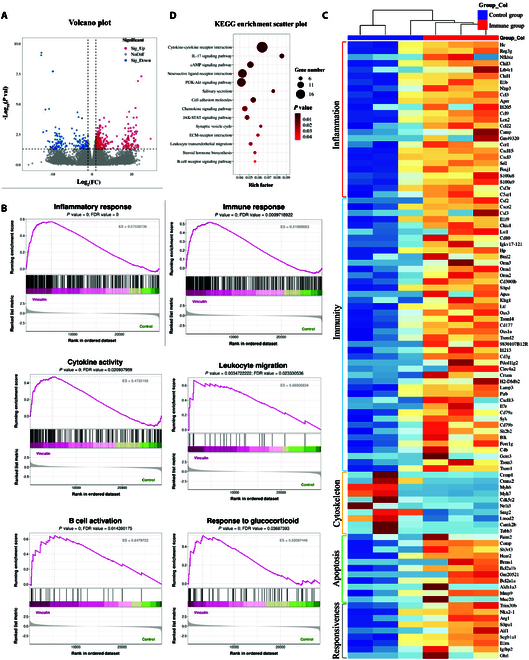
Transcriptome analysis of renal tissues from control and vinculin actively immunized mice. (A) Volcano plot of DEGs (vinculin-immunized versus control). Red dots, up-regulated genes; blue dots, down-regulated genes; gray dots, nonsignificant genes. (B) GSEA enrichment score (ES) curve for the control and vinculin-immunized groups. The ES curve shows scores at each position, with the peak score indicating gene set enrichment. The dashed lines mark the maximum/minimum ES values and gene positions. The black lines indicate gene positions in the ranked list. Heatmap colors: red represents high expression in the immunized group, green represents the control group, and gray represents the signal-to-noise ratio. (C) Heatmap of selected DEGs in control versus vinculin-immunized mice. The samples on the *x* axis and the DEGs on the *y* axis are shown. The color gradient from blue (low expression) through white to red (high expression) indicates gene expression levels. (D) Bubble chart of the KEGG enrichment results. The *x* axis (rich factor) shows the ratio of DEGs in a pathway to total genes, with larger bubbles indicating more enriched genes. Pathways are listed on the *y* axis. The bubble color indicates the *P* value, reflecting statistical significance.

Renal tissues procured from vinculin-immunized mice and control mice were subjected to comprehensive transcriptome sequencing. We screened for differentially expressed genes (DEGs) using thresholds of a fold change ≥ 2 (i.e., absolute log2FC ≥ 1) and a *P* value < 0.05 (|log2FC| ≥ 1 and *P* < 0.05). The results revealed 387 up-regulated genes and 134 down-regulated genes between the immune group and the control group. The differential expression profiles of these DEGs were visualized through a volcano plot (Fig. 7A). To elucidate the biological significance and potential mechanistic underpinnings associated with the identified DEGs, we conducted gene set enrichment analysis (GSEA). The results revealed notable enrichment in gene sets related to inflammatory processes, immune responses, cytokine activities, leukocyte trafficking, B cell activation, and hormonal sensitivities (Fig. 7B). Complementing these findings, Gene Ontology (GO) enrichment analysis confirmed that the enrichment pathways linked to “inflammation-related processes”, “immunity-related pathways”, “apoptosis and autophagy”, and “responsiveness to glucocorticoids” were up-regulated. In contrast, pathways associated with "cytoskeletal functions" were down-regulated. The expression levels of pivotal genes within these pathways are presented in the heatmap (Fig. 7C). Moreover, the results of Kyoto Encyclopedia of Genes and Genomes (KEGG) pathway enrichment analysis mirrored these observations (Fig. 7D).

## Discussion

Identifying early diagnostic markers and therapeutic targets to achieve early diagnosis, precise classification, and personalized treatment is a crucial research direction in autoimmune diseases [23–25]. In this study, we found that anti-vinculin autoantibody levels were specifically elevated in INS patients and significantly correlated with disease activity, underscoring their potential relevance in assessing disease severity and activity. Longitudinal observations further revealed a decrease in these autoantibody levels during clinical remission, supporting their potential utility as dynamic biomarkers for assessing treatment response and predicting relapse. Most pediatric patients with INS respond well to glucocorticoid therapy and have a good prognosis [26,27]. However, frequent relapses, steroid dependence or resistance, and insensitivity remain in some patients, who are more likely to develop kidney failure [28–30]. The elevated prevalence of anti-vinculin autoantibodies in steroid-resistant forms of INS, particularly among patients with MCD and FSGS, further supports a possible role in mediating or reflecting resistance to conventional immunosuppressive therapies.

To better understand the pathogenic potential of anti-vinculin autoantibodies in renal injury, we employed both passive and active immunization strategies in mouse models. The passive transfer of anti-vinculin antibodies led to transient proteinuria, and the temporary nature of this response underscores the limitations of such models in replicating the sustained and multifaceted immune activation observed in human disease. This approach, although useful for demonstrating short-term effects of circulating antibodies, does not encompass the full spectrum of autoimmune pathology, particularly continuous autoantibody production and interplay with cellular immune components that characterize chronic immune-mediated glomerular diseases. In contrast, active immunization with recombinant vinculin antigen offers a more physiologically relevant model by stimulating endogenous anti-vinculin autoantibody production. The resulting phenotype closely resembled the clinical and pathological features of immune-mediated podocytopathies, including persistent proteinuria, hypoalbuminemia, and podocyte injury, as evidenced by IgG deposition and foot process effacement. These findings suggest that sustained anti-vinculin immune responses may directly contribute to podocyte dysfunction, either through antibody-mediated damage or by triggering downstream inflammatory and structural remodeling pathways. Compared with those from control mice, transcriptomic profiling of renal tissues from vinculin-immunized mice revealed significant alterations in gene expression. The enrichment analyses consistently highlighted the activation of inflammatory and immune-related pathways, including cytokine signaling, leukocyte trafficking, and B cell activation, as well as pathways linked to apoptosis, autophagy, and glucocorticoid responsiveness. Conversely, genes involved in cytoskeletal organization were notably down-regulated. These findings provide compelling evidence that immunization with vinculin triggers a broad immunoinflammatory response in the kidney, potentially contributing to tissue injury and podocyte dysfunction. The down-regulation of cytoskeletal pathways may reflect structural alterations within glomerular cells, which is consistent with impaired glomerular barrier function. Furthermore, the up-regulation of glucocorticoid-responsive pathways suggests that autoantibody-mediated inflammation may influence treatment responsiveness.

Over the past few years, significant progress has been made in understanding the role of autoantibodies targeting podocytes in the pathogenesis of INS. Specifically, autoantibodies against nephrin [2,6,7,12], crb2 [9], annexin A2 [16], PSMA1 [18], and UCHL1 [8] have emerged as pivotal players, offering new possibilities for targeted and effective therapies [1]. Studies have highlighted the importance of these autoantibodies in disease progression and relapse. For instance, Watts et al. [7], Shirai et al. [12], and Hengel et al. [2] provided compelling evidence that anti-nephrin autoantibodies are associated with disease activity in both MCD and recurrent FSGS post-kidney transplantation, suggesting that autoantibody profiles could serve as potential biomarkers for disease monitoring and prognosis. In addition to anti-nephrin autoantibodies, other researchers have proposed autoantibodies targeting alternative podocyte proteins. Jamin et al. [8] reported that anti-UCHL1 autoantibodies are closely related to the recurrence of INS. Hada et al. [9] identified anti-Crb2 autoantibodies and successfully established an immunized mouse model with Crb2. Raglianti et al. [13] identified anti-podocin and anti-kirrel1 autoantibodies as highly specific biomarkers for active INS. Our research team has identified 10 distinct podocyte autoantibodies, including anti-annexin A2 [16], anti-PSMA1 [18], anti-vinculin autoantibodies, and so on. Among these, anti-vinculin autoantibodies warrant particular attention because of their potential role in disrupting the structural integrity of podocytes. Vinculin is a critical component of the podocyte cytoskeleton and is involved in maintaining cell adhesion and mechanical stability. Anti-vinculin autoantibodies may interfere with the interaction of vinculin with integrins and actin filaments, thereby impairing podocyte foot process architecture and slit diaphragm function. By integrating these recent insights, our findings on anti-vinculin autoantibodies provide a more comprehensive picture of how autoantibodies contribute to INS pathogenesis.

From a broader perspective, these findings contribute to the growing body of evidence that supports a paradigm shift in understanding INS, not merely as a glomerular disorder with unknown etiology but also as a disease potentially driven by pathogenic autoantibodies. Recognizing INS as an antibody-mediated disease has meaningful clinical implications: It encourages the development of immunologically targeted diagnostics and therapies, and it opens new possibilities for earlier identification of high-risk patients through noninvasive serologic screening. Such an approach could move clinical practice away from the prevailing trial-and-error strategy, especially in predicting steroid responsiveness, and toward more personalized, mechanism-based treatment algorithms [31]. While anti-vinculin autoantibodies may not act in isolation, their role, either as pathogenic contributors or as biomarkers of immune activity, warrants further mechanistic investigation. The interaction of these proteins with other podocyte-specific autoantibodies and their downstream effects on glomerular function remain important areas for future research. Nonetheless, their identification adds valuable information to the complex immunological puzzle of immune-mediated podocytopathies.

Our findings highlight the potential pathogenic role of anti-vinculin autoantibodies in podocyte injury in INS, supporting the broader hypothesis that certain forms of nephrotic syndrome may represent antibody-driven autoimmune diseases. However, several limitations should be acknowledged. First, the clinical cohort in this preliminary study was relatively small. To address this, we are currently conducting a large-scale, multicenter national trial to rigorously assess and confirm the clinical relevance of a panel of podocyte-targeted autoantibodies, including anti-vinculin autoantibodies, with the aim of enhancing early diagnostic accuracy and facilitating precise phenotypic classification of INS. Second, while we have demonstrated the presence and pathogenic potential of anti-vinculin autoantibodies through both human data and experimental models, the initial triggers of the autoimmune response and the mechanisms governing autoantibody production remain poorly understood. Future research will focus on integrating the exploration of immunoregulatory pathways with an in-depth analysis and exploring how genetic predisposition and environmental exposures interact to drive the development of these podocyte-specific autoantibodies. Third, a fundamental and unresolved question concerns the targeting of vinculin, an intracellular protein, by circulating autoantibodies. How these antibodies access intracellular compartments and exert their pathogenic effects remains unclear, representing a critical area for future mechanistic investigation.

In conclusion, while important questions remain, our findings lay the groundwork for a paradigm shift in how INS is understood and managed. Anti-vinculin autoantibodies may serve as noninvasive biomarkers for diagnosis, risk stratification, and real-time monitoring of disease activity and treatment response. This could significantly reduce the reliance on renal biopsy and minimize delays in therapeutic decision-making. Understanding the mechanisms underlying anti-vinculin antibody generation and function could facilitate the development of novel therapeutics, such as antigen-specific immunomodulation or blocking antibodies that prevent interactions with critical intracellular targets. Continued translational research in this area will be essential to fully harness the diagnostic and therapeutic potential of this emerging biomarker.

## Methods

### Clinical samples

A total of 147 patients who fulfilled the consensus-based diagnostic criteria for nephrotic syndrome and whose serum samples were available were recruited from the Children’s Hospital of Zhejiang University between 2020 September 1 and 2021 September 30. Nephrotic syndrome was defined as significant proteinuria (>50 mg/kg/day, or urinary protein/creatinine ratio > 2.0, or 3+ to 4+ on urine dipstick 3 times in 1 week) and hypoalbuminemia (<30 g/l). Patients with secondary nephrotic syndrome due to infections, malignancies, medications, hereditary conditions, or other associated disorders [such as lupus nephritis and IgA nephropathy (IgAN)] were excluded. Eighty-four healthy controls were randomly selected from blood donors under 18 years of age who had no proteinuria or history of nephrotic syndrome. Additionally, 100 patients each with IgAN, Henoch–Schönlein purpura nephritis (HSPN), Henoch–Schönlein purpura (HSP), and Kawasaki disease (KD) were included as disease control groups. The study population is illustrated in Fig. 1A and Fig. S1. The Ethics Committee of the Children’s Hospital, Zhejiang University School of Medicine (2021-IRB-031), approved this study.

### Protein microarray for anti-vinculin autoantibody detection

Recombinant human vinculin protein was custom synthesized by GenScript, USA (lot number: C6602HA190-35/P5HD001), with a concentration of 2.20 mg/ml (determined by Bradford) and a purity exceeding 90% [verified by sodium dodecyl sulfate–polyacrylamide gel electrophoresis (SDS-PAGE) under reducing conditions]. Vinculin (60 μg/ml) was spotted onto a nitrocellulose membrane (0.8-μm pore size, Sartorius, Germany) through a chip sampling apparatus (AD1500, BioDot). Biotin-labeled mouse anti-human IgG (Thermo Fisher) and inactivated serum (56 °C) served as positive and negative controls, respectively. The nitrocellulose membrane was incubated for 1 h in 5% bovine serum albumin (BSA) blocking solution. Undiluted patient serum (300 μl) was added and incubated for 45 min, followed by washing with Tris buffer. A biotin-conjugated anti-human IgG antibody complex (300 μl) was then added and incubated for 45 min. After another wash, an enzyme mixture (300 μl) was added, and the mixture was incubated at room temperature for 20 min. Following a repeat wash, substrate solution (300 μl) was added for color development, and the mixture was incubated for 20 min. The reaction was stopped with tap water. After drying, the grayscale value was measured.

### Injection of anti-vinculin antibodies into mice

The purified anti-vinculin polyclonal antibody was custom-made by Wuhan Dia-An Biotechnology Co. Ltd. (catalog number: s2486-1), with a concentration of 7 mg/ml, and was free of glycerol and sodium azide. Female BALB/c wild-type mice, aged 6 to 8 weeks and weighing between 18 and 22 g, were selected as the immunization subjects. The mice in the experimental group received a tail vein injection of 1,000 μg of anti-vinculin antibodies, whereas those in the control group were injected with an equal volume of the IgG isotype control antibody. Sera were collected at 3 critical time points: 24, 96, and 168 h post-injection, to dynamically monitor the antibody levels in vivo. For 1 week following the initial administration, daily urine samples were collected to assess urinary albumin and creatinine levels. One week after the antibody injection, the mice were euthanized, and their kidneys were excised for detailed histological analysis and ultrastructural examination under an electron microscope.

### Active immunization of mice with vinculin protein

Recombinant murine vinculin protein was custom synthesized by HUABIO, China (lot number: TP40896), with a concentration of 1.2 mg/ml (by A280) and a purity exceeding 90% (SDS-PAGE under reducing conditions). The buffer was replaced with phosphate-buffered saline (PBS). The vinculin protein was diluted in PBS to a concentration of 1 mg/ml. An antigen–adjuvant emulsion was then formulated by vigorously blending the diluted antigen with an equal volume of Freund’s complete adjuvant (Sigma-Aldrich). The emulsion (containing 100 μg of vinculin protein) was administered to the BALB/c wild-type mice. For booster immunizations, the antigen (0.5 mg/ml) was emulsified with Freund’s incomplete adjuvant at a 1:1 volume ratio, with the same injection volume per mouse. A total of 4 booster immunizations were administered. Antibody titers were quantified in the blood serum of the mice via ELISA following each immunization round. Periodic urine collection facilitated the quantitative analysis of urinary protein. At specified intervals, the mice were euthanized, and kidney tissues were dissected for analysis via histopathological staining and electron microscopy to assess tissue damage. The strategies for active immunization with recombinant vinculin protein are illustrated in Fig. 5A.

### Detection of vinculin autoantibody titers in mouse serum

The recombinant vinculin antigen (1 μg/ml in coating buffer, Na_2_CO_3_ and NaHCO_3_) was added to a 96-well plate (50 μl/well) and incubated overnight at 4 °C. The buffer was discarded, and the wells were washed 3 times with 1× Tris-buffered saline with Tween 20 (TBST), blocked with 60 μl of 1% BSA (in TBST) at 37 °C for 1 h, and then incubated with 1:10 diluted serum samples (50 μl/well, centrifuged at 3,000 rpm for 5 min) at 37 °C for 1 h. Positive (positive serum) and negative (1% BSA) controls were included. After washing, a 1:5,000 diluted horseradish peroxidase (HRP)-conjugated secondary antibody (50 μl/well) was added, followed by a 45-min incubation at 37 °C. The wells were washed again, and 100 μl/well of tetramethylbenzidine (TMB) substrate solution was added for 5 min at 37 °C. The reaction was stopped with 90 μl/well of 2 M sulfuric acid, and the absorbance was measured at 450 nm via a microplate reader.

### Detection of urinary albumin and creatinine

Urinary albumin was quantified via a mouse albumin detection ELISA kit (Chondrex Inc., catalog no. 3012). Urinary creatinine was quantified via a creatinine colorimetric assay kit (Cayman, item no. 500701). Both procedures were performed according to the manufacturer’s protocols. The urinary albumin-to-creatinine ratio was subsequently calculated.

### Kidney histology

Kidneys were washed with prechilled PBS to remove residual blood and then fixed in 4% paraformaldehyde for 24 h. After fixation, they were dehydrated through an ethanol gradient (85%, 90%, and 100%) and rendered transparent in xylene twice for 10 min each. The kidneys were embedded in paraffin, which was quickly hardened upon cooling. The paraffin blocks were sectioned into 3- to 5-μm slices via a microtome. After drying, the sections were stained with H&E, PAS, and Masson’s trichrome. The stained sections were examined and photographed under a regular light microscope.

### Kidney immunofluorescence

Five-micrometer-thick frozen kidney tissue sections were fixed in 4% paraformaldehyde for 30 min and permeabilized with 0.4% Triton X-100 in PBS for 20 min. After being washed with PBS, the sections were blocked with 5% BSA in PBS for 1 h at room temperature. Primary antibodies diluted in 1% BSA-PBS were applied overnight at 4 °C. The next day, the sections were incubated with secondary antibodies for 1 h at room temperature in the dark. After being washed, the sections were stained with 4′,6-diamidino-2-phenylindole (DAPI) (Beyotime, C1002) for 10 min at room temperature and washed again 3 times. An anti-fluorescence quencher was applied to protect the fluorescence, and coverslips were placed on the slides. High-resolution images were captured via a Leica TCS-SP8 confocal laser scanning microscope with 20× and 40× objectives. Details of the primary and secondary antibodies used for immunofluorescence and immunohistochemical staining are provided in the Supplementary Materials.

### TEM and SEM

Kidney samples were fixed in phosphate-buffered 2.5% glutaraldehyde (pH 7.4) and then in 1% osmium tetroxide for 1 to 1.5 h. For TEM, kidney tissues were stained with 2% uranyl acetate (100 μl, 30 min at room temperature), dehydrated through graded ethanol and 100% acetone, and embedded. Ultrathin sections were cut using a Leica UC7, followed by staining and positioning. Observation and imaging were performed on a Tecnai G2 Spirit 120-kV cryo-transmission electron microscope. For SEM, the kidney tissue blocks were dehydrated directly via a gradient of alcohol after osmium tetroxide fixation. After critical point drying and coating, the samples were observed and photographed via a Nova Nano 450 field emission scanning electron microscope.

### RNA extraction and library construction

Total RNA was extracted from mouse kidney samples via TRIzol reagent (Invitrogen, Carlsbad, CA, USA) and quantified with a NanoDrop ND-1000 (NanoDrop, Wilmington, DE, USA). RNA integrity was assessed by a Bioanalyzer 2100 (Agilent, CA, USA), with an RNA integrity number (RIN) of >7.0, and confirmed by electrophoresis on a denaturing agarose gel. Poly(A) RNA was purified from 1 μg of total RNA via Dynabeads Oligo (dT) 25-61005 (Thermo Fisher, CA, USA) and fragmented with the Magnesium RNA Fragmentation Module (NEB, catalog no. e6150, USA) at 94 °C for 5 to 7 min. The RNA fragments were reverse transcribed to cDNA by SuperScript II Reverse Transcriptase (Invitrogen, catalog no. 1896649, USA) and converted to U-labeled second-strand DNAs with *Escherichia coli* DNA polymerase I (NEB, catalog no. m0209, USA), RNase H (NEB, catalog no. m0297, USA), and dUTP Solution (Thermo Fisher, catalog no. R0133, USA). After an A-base was added to the blunt ends, the indexed adapters were ligated, and size selection was performed via AMPureXP beads. The U-labeled second strands were digested with heat-labile UDG enzyme (NEB, catalog no. m0280, USA), and the libraries were polymerase chain reaction (PCR)-amplified (initial denaturation at 95 °C for 3 min; 8 cycles of denaturation at 98 °C for 15 s, annealing at 60 °C for 15 s, and extension at 72 °C for 30 s; and a final extension at 72 °C for 5 min). The final library had an average insert size of 300 ± 50 base pairs (bp). Sequencing was performed on an Illumina NovaSeq 6000 (LC-Bio Technology Co. Ltd., Hangzhou, China) via 2 × 150 bp paired-end sequencing (PE150).

### Bioinformatic analysis of RNA-seq data

Fastp software (https://github.com/OpenGene/fastp) was used to remove the reads that contained adaptor contamination, low-quality bases, and undetermined bases with the default parameters. The sequence quality was subsequently verified via fastp. We used HISAT2 (https://ccb.jhu.edu/software/hisat2) to map reads to the reference genome of *Homo sapiens* GRCh38. The mapped reads of each sample were assembled via StringTie (https://ccb.jhu.edu/software/stringtie) with default parameters. All the transcriptomes from all the samples were subsequently merged to reconstruct a comprehensive transcriptome via gffcompare (https://github.com/gpertea/gffcompare/). After the final transcriptome was generated, StringTie was used to estimate the expression levels of all the transcripts. StringTie was used to determine the expression levels of mRNAs by calculating the fragments per kilobase of transcript per million mapped reads (FPKM). FPKM = [total_exon_fragments/mapped_reads(millions) × exon_length(kB)]. The differentially expressed mRNAs with a fold change > 2 or < 0.5 and a parametric *F* test comparing nested linear models (*P* < 0.05) were selected via the R package edgeR. Finally, gene enrichment analysis for GO and KEGG pathways was conducted via the DAVID bioinformatics resource (https://david.ncifcrf.gov/).

### Western blotting

Protein extraction was achieved by lysing the cells in radioimmunoprecipitation assay (RIPA) buffer (Thermo Fisher) supplemented with a protease inhibitor cocktail (MedChemExpress). The total protein content was then quantified to ensure equal loading. The extracted proteins were subjected to heat-induced denaturation at 96 °C for 5 min to disrupt tertiary structures and linearize polypeptide chains. Following denaturation, the proteins were resolved via SDS-PAGE on 10% gels. The proteins were subsequently electrotransferred onto polyvinylidene difluoride (PVDF) membranes. To minimize nonspecific binding, the membranes were blocked with a solution of nonfat milk in TBST. Overnight incubation at 4 °C with primary antibodies was conducted to specifically bind target proteins. After thorough washing to remove unbound primary antibodies, the membranes were probed with HRP-conjugated secondary antibodies. The bound antibodies were detected via enhanced chemiluminescence (ECL) reagents (Biosharp), followed by exposure to chemiluminescence film for visualization.

### Statistical analysis

In this study, categorical variables were compared via chi-square tests, while continuous variables were assessed on the basis of their distribution. Normally distributed data are presented as the means ± standard deviations (SDs), and non-normally distributed data are presented as medians with ranges. For pairwise comparisons between groups, least significant difference (LSD) tests were used, whereas one-way analysis of variance (ANOVA) was applied for comparisons among multiple independent normally distributed groups. The Mann–Whitney *U* test was used to compare non-normally distributed continuous variables. *P* < 0.05 was considered to indicate statistical significance. *P < 0.05, **P < 0.01, ***P < 0.001, ****P < 0.0001. All the statistical analyses were performed with GraphPad Prism version 9.5.1.

## Data Availability

All the resources generated in this study are available upon request from the lead author upon providing a completed material transfer agreement.
