# Supplementary material for: Autoantibodies Targeting Vinculin Reveal Novel Insight into the Mechanisms of Autoimmune Podocytopathies
Source: Research (Wash D C). 2025 Jun 3;8:0722. doi: 10.34133/research.0722 (PMC12131845; doi:10.34133/research.0722)
Supplement: Supplementary 1 — Figs. S1 to S3 Table S1 [file research.0722.f1.docx]

**SUPPLEMENTARY MATERIAL**

**Autoantibodies Targeting Vinculin Reveal Novel Insight into the Mechanisms of Autoimmune Podocytopathies**

**Table. S1 Antibodies used for immunofluorescence and immunohistochemistry staining**

| **Brand** | **Antibody name** | **Item number** |
| --- | --- | --- |
| Proteintech | Vinculin Mouse McAb | 66305-1-Ig |
| Proteintech | Vinculin Rabbit PolyAb | 26520-1-AP |
| Proteintech | SYNPO Monoclonal antibody | 67339-1-Ig |
| Proteintech | SYNPO Polyclonal antibody | 21064-1-AP |
| Abcam | Anti-Synaptopodin antibody | ab224491 |
| Invitrogen | Goat anti-Human IgG (H+L) Cross-Adsorbed Secondary Antibody, Alexa Fluor™ 488 | A-11013 |
| Invitrogen | Goat anti-Rabbit IgG (H+L) Cross-Adsorbed Secondary Antibody, Alexa Fluor™ 594 | A-11012 |
| Invitrogen | Goat anti-Mouse IgG (H+L) Cross-Adsorbed Secondary Antibody, Alexa Fluor™ 647 | A-21235 |

**
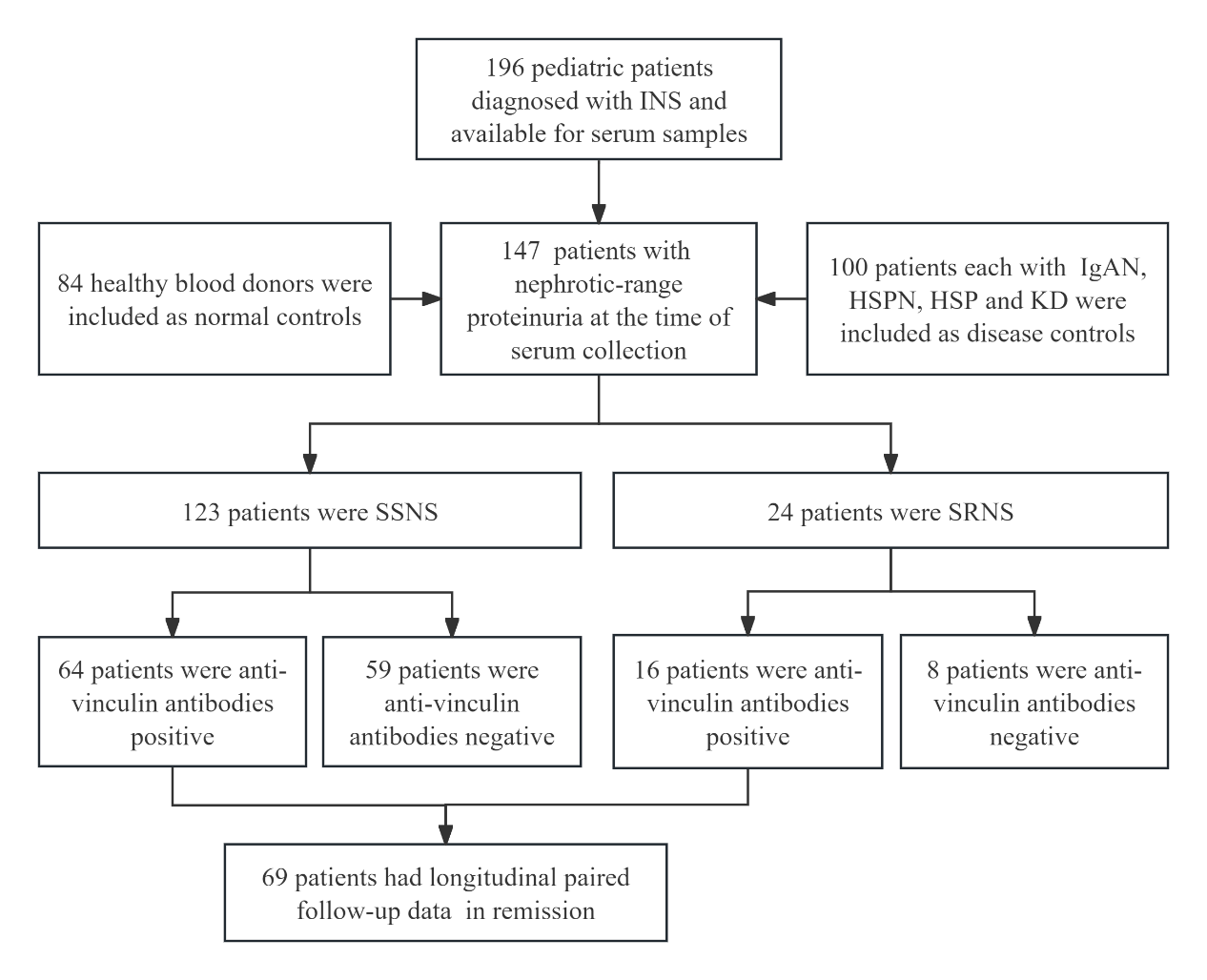
**

**Fig. S1 Flow chart for the selection of the study population**

IgAN: IgA nephropathy; HSPN: Henoch-Schönlein purpura nephritis; HSP: Henoch-Schönlein purpura; KD: Kawasaki disease; SSNS: Steroid-sensitive nephrotic syndrome; SRNS, Steroid-resistant nephrotic syndrome


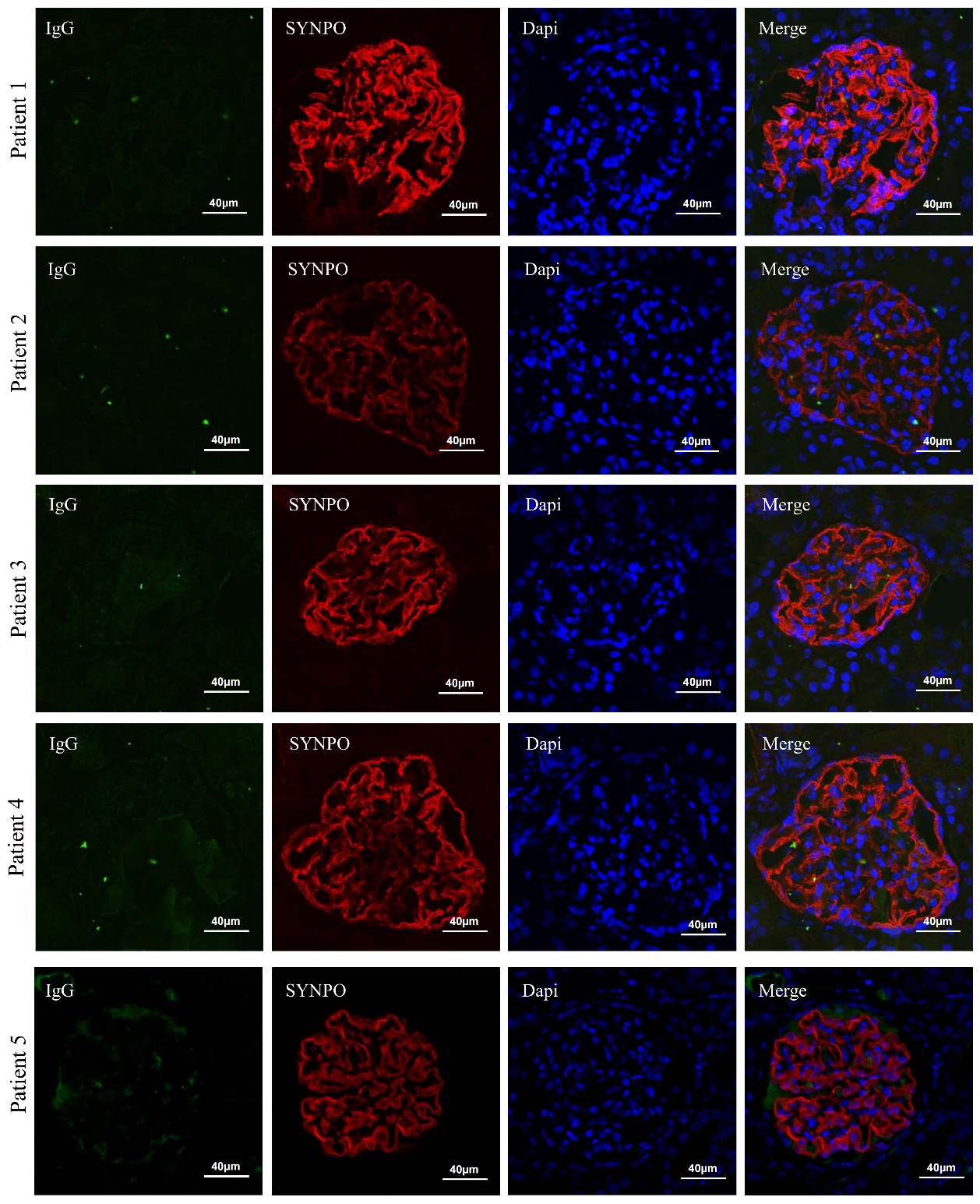


**Fig. S2 IgG deposition in human kidneys.** Immunofluorescence visualization of IgG deposition in paracancerous normal renal tissue (patient 1) and other kidney disease samples, including IgA nephropathy (patient 2 & 3) and Henoch-Schönlein purpura nephritis (patient 4 & 5). Magnification: 60× (scale bar = 40 µm).


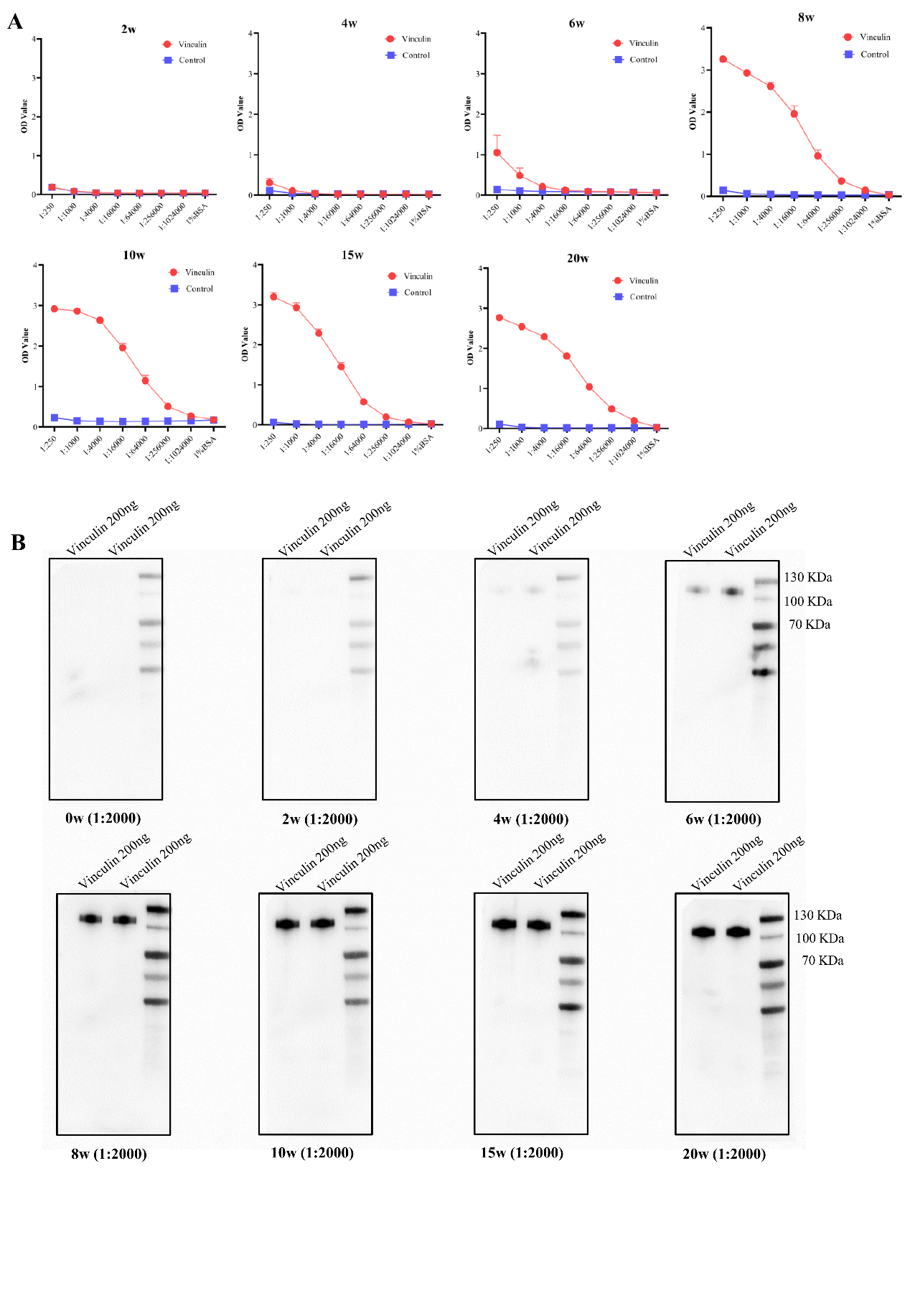


**Fig. S3 Active immunization induced the production of antivinculin autoantibodies.** (A) Titers of antivinculin autoantibodies in mouse serum at various time points (2nd week, 4th week, 6th week, 8th week, 10th week, 15th week, and 20th week) following initial active immunization with vinculin. The red line represents the group actively immunized with vinculin, whereas the blue line represents the control group. (B) Western blot protein electrophoresis strip diagram to evaluate the immune effect of active immunization with recombinant murine vinculin.
